# Fine structure of sensory apparatus on the head of *Cixiopsis punctatus*

**DOI:** 10.1093/jis/14.1.99

**Published:** 2014-07-22

**Authors:** Rong-rong Wang, Xin-yi Wan, Ai-ping Liang

**Affiliations:** Key Laboratory of Zoological Systematics and Evolution, Institute of Zoology, Chinese Academy of Sciences, 1 Beichen West Road, Chaoyang District, Beijing 100101, P.R. China

**Keywords:** Hemiptera, Tropiduchidae, antenna, maxillae, labium, ultrastructure

## Abstract

The external morphology of the heads of adult male and female *Cixiopsis punctatus* (Matsumura) (Hemiptera: Fulgoromorpha: Tropiduchidae) was studied using scanning electron microscopy. Eleven types of sensilla or sensory organs were identified: trichoid sensilla on the pedicel, scape, maxillae, and labium; campaniform sensilla on the antennal pedicel, antennal scape, maxillae, and labium; plate organs on the antennal pedicel; coeloconic sensilla in Bourgoin’s organ and styloconic sensilla on the expanded flagellar base; Evans’ organ and button-like sensilla on the maxillary plates; basiconic sensilla, peg sensilla, and coin-shaped sensilla on the labium. Styloconic sensilla on the expanded flagellar base and peg sensilla located between the dorsal sensory field and the opening of the maxillae and mandibles were first reported in Tropiduchidae. The external morphology, distribution, and abundance of sensilla located on antennae, maxillae, and labium in *C. punctatus* were illustrated.

## Introduction

The Fulgoromorpha, commonly named planthoppers, constitute a large group of phytophagous insects in the order Hemiptera, including about 14,000 described species and 30 recent or fossil families worldwide. It is an old group of Hemiptera, known from fossils of the lower Permian (ca. 258 million years ago), and their phylogeny is still not well understood ( Bourgoin 2013 ).

Although molecular characters are now widely used to reconstruct the phylogeny of Fulgoromorpha ( Bourgoin et al. 1997 ; Yeh et al. 1998 , 2005; Yeh and Yang 1999; Bourgoin and Campbell 2002 ; Urban and Cryan 2007 ; Song and Liang 2013 ) and to test existing phylogenetic hypotheses ( Muir 1923 , 1930; Asche 1987 ; Emeljanov 1990 ; Bourgoin 1993 ; Chen and Yang 1995 ), several recent studies have also used morphological characters with varying degrees of success ( Bourgoin and Deiss 1994 ; Hamilton 2011 ; Brożek and Bourgoin 2012 ). Evidence that morphology is a source of information is far from exhausted in Fulgoromorpha systematics.

Based on studies of comparative morphology ( Bugnion 1908 ) and ultrastructure ( Lewis and Marshall 1970 ; Stroiński et al. 2011 ; Brożek and Bourgoin 2012 ) of different antennal (plate organs, Bourgoin's organ), labial (labial sensilla), and maxillary (Evans’ organ) sensillae of the Fulgoromorpha, a remarkable disparity in the same sensory equipment is observed, which has value for taxonomic and phylogenetic analyses ( Bourgoin 1986 ; Bourgoin and Deiss 1994; Liang 2001 ; Hamilton 2011 ; Stroiński et al. 2011 ; Brożek and Bourgoin 2012 ). However, there is still a paucity of the anatomical data on Fulgoromorpha, let alone the anatomy of Tropiduchidae.

The family Tropiduchidae is the keystone group for understanding evolutionary processes within the higher Fulgoroidea. Our present state of knowledge does not allow unambiguous indication of the ancestral group for the tropiduchids, and thereby at least part of the higher Fulgoroidea. Fennah (1982) revised the higher classification of the Tropiduchidae and recognized 15 tribes in the family. The genus *Cixiopsis* was included in the tribe Cixiopsini. Subsequently, this study investigated, through scanning electron microscopy (SEM) observations, sensory equipment on the head of *Cixiopsis punctatus* (Matsumura) (Hemiptera: Fulgoromorpha: Tropiduchidae) as potential sources for new characters for future comparative morphological studies in Tropiduchidae and Fulgoromorpha.

## Materials and Methods

The external morphology of adult *C. punctatus* (one male and three females) was studied using SEM. All the samples were obtained from Fujian Province, China, and were deposited in the Institute of Zoology, Chinese Academy of Sciences, Beijing, China (IZCAS). For SEM observation, the heads together with antennae were first removed from the specimens. They were then cleaned in a chloroform bath or a lukewarm 10% KOH bath in an ultrasonic cleaner (1 min), followed by twice cleaning in 75% alcohol (2 min for each case) to remove the cuticular waxy powder on the samples’ surface. They were then dehydrated in a graded ethanol series, after which they were dried at critical point drying, mounted on stubs with double-sided adhesive tape, and coated with a film of gold-palladium. Finally, observations were made with a Hitachi S34Q SEM ( www.hitachi.com ) at the Microscopy Core Facility, Biological Technology Center, Beijing Forestry University. Terminology for the antennal sensilla description follows Bourgoin and Deiss ( 1994 ) and Wang et al. (2013) .

## Results

### General description of the antenna

**Figure 1.**
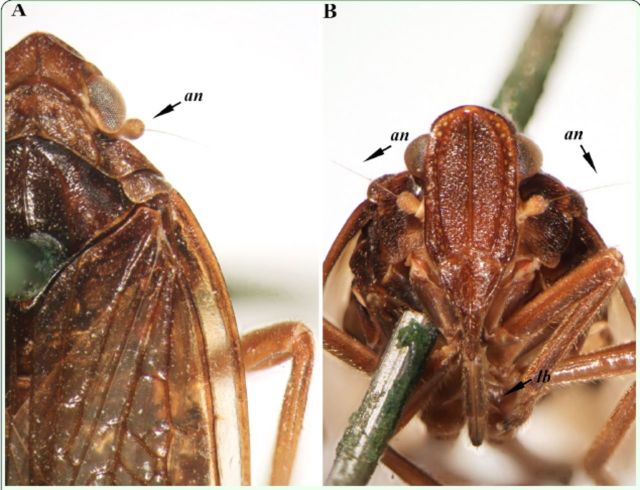
General views of the antennae and labium of *Cixiopsis punctatus* . A: Dorsal view of the head, showing an antenna (an). B: Ventral view of the head, showing two antennae (an) and the labium (lb). High quality figures are available online

**Figure 2.**
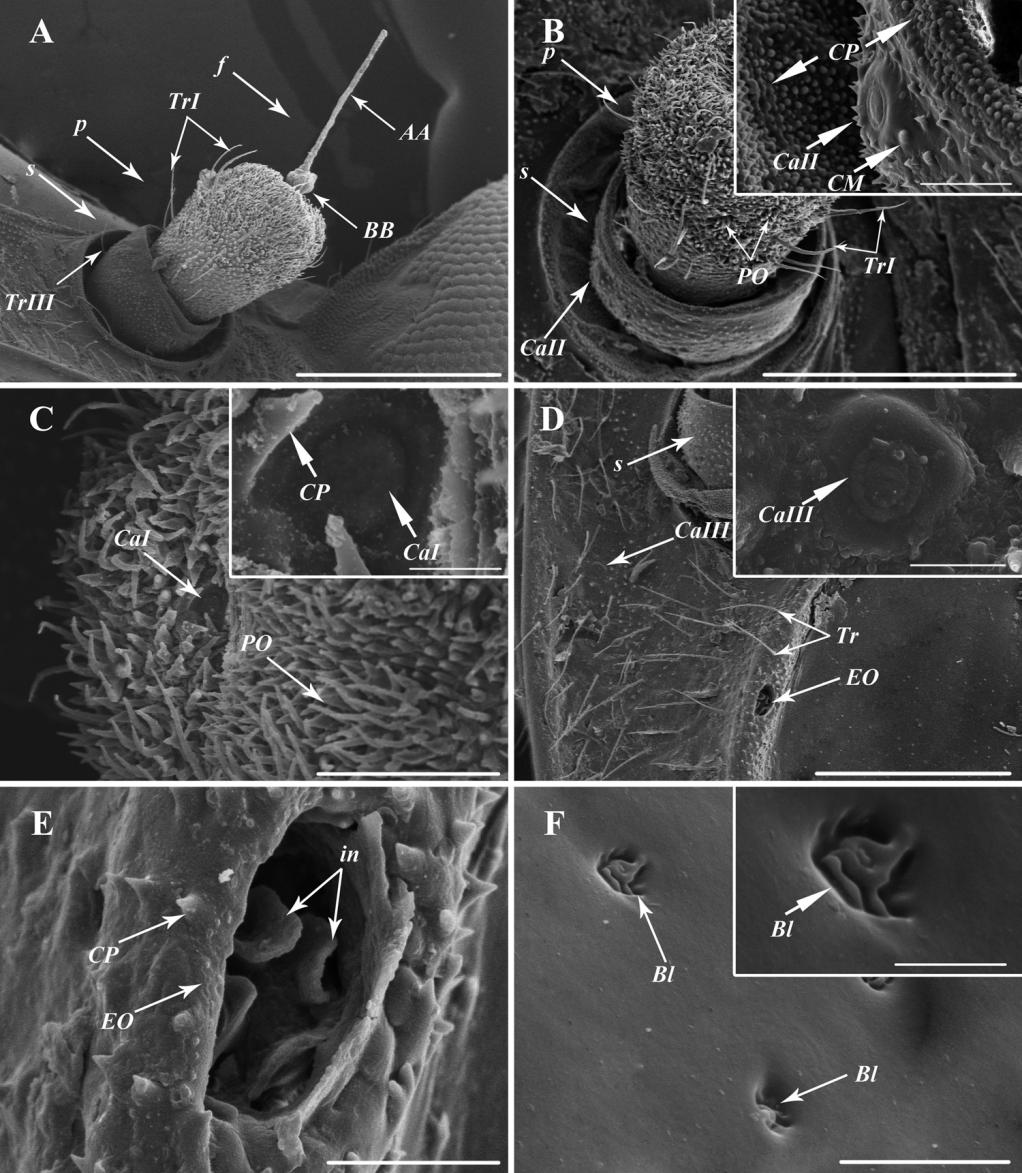
SEM images of *Cixiopsis punctatus.* A: General view of the antenna showing the antennal scape (s) with trichoid sensilla subtype III (TrIII), antennal pedicel (p) with trichoid sensilla subtype I (TrI) and the antennal flagellum (f) including the apical arista (AA) and basal bulb (BB). B: General view of the campaniform sensilla subtype II (CaII) and cuticular microtubercles (CM) on the antennal scape (s), the trichoid sensilla subtype I (TrI) and plate organs (PO) on the antennal pedicel (p). C: The top of the antennal pedicel, showing the campaniform sensilla subtype I (CaI), the plate organs (PO) and the cone-shaped processes (CP). D: Maxillary plate under the antennal scape, showing the Evans’ organ (EO), trichoid sensilla (Tr) and campaniform sensilla subtype III (TrIII). E: Enlarged view of the Evans’ organ (EO), with infoldings (in) inside. F: Button-like sensilla (Bl) on the maxillary plate. [(A) scale bar 300 **|x** m, (B) scale bar 200 **|x** m, 20 **|x** m in box, (C) scale bar 50 **|x** m, 5 **|x** m in box, (D) scale bar 200 **|x** m, 10 **|x** m in box, (E) scale bar 20 **|x** m, (F) scale bar 10 **|x** m, 3 **|x** m in box]. High quality figures are available online.

**Figure 3.**
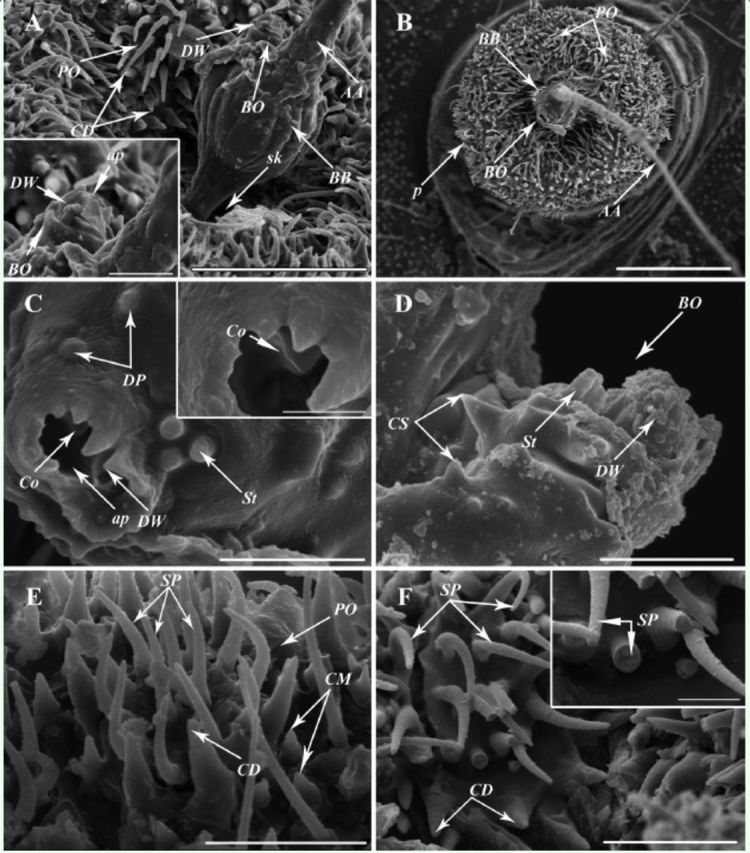
SEM images of the antennal pedicel and antennal flagellum of *Cixiopsis punctatus.* A: Bourgoin’s organ (BO) surrounded by denticlelike walls (DW) on the flagellar basal bulb (BB), which is inserted into a socket (sk). B: General view of the antenna showing the Bourgoin's organ (BO). C: Top view of the Bourgoin's organ (BO) showing a coeloconic sensillum (Co), an aperture (ap), three styloconic sensilla (St), denticlelike walls (DW) and dome-like processes (DP). D: Lateral view of the Bourgoin’s organ (BO), showing the cuticular spines (CS), styloconic sensilla (St) and denticlelike walls (DW). E: Plate organs (PO) with setalike projections (SP), surrounded by cuticular denticles (CD) and cuticular microtubercles (CM). F: Solid setalike projections (SP) and cuticular denticles (CD). [(A) scale bar 50 **|x** m, 10 **|x** m in box, (B) scale bar 100 **|x** m, (C) scale bar 10 **|x** m, 5 **|x** m in box, (D) scale bar 10 **|x** m, (E) scale bar 20 **|x** m, (F) scale bar 20 **|x** m, 5 **|x** m in box]. High quality figures are available online.

In *C. punctatus,* antennae are situated on the lateral region of the head capsule beneath the compound eyes, as in other planthoppers ( Figure 1 ). In both male and female, each antenna is about 1268 µm long and consists of three segments: a short basal scape, a cylindrical pedicel, and a thread-like flagellum ( Figure 2A ). The scape is about 59-108 µm long and directly attached to the head capsule, bearing few sensilla ( Figure 2A , B). The antennal pedicle is about 230 µm long and is covered by numerous trichoid sensilla and plate organs (Figures 2B, 3B), on the top of which a campaniform sensillum is revealed ( Figure 2C ). The flagellum (about 955 µm in length) is composed of two distinct portions: a basal bulb with a short petiole at the extreme base and an apical arista ( Figure 2A ). The basal bulb, a swollen base of the flagellum, is proximally inserted on the pedicel at the level of a disk-like area ( Figure 3A ). The area is encircled by concentrically arranged cuticular spines ( Figure 3A ). On the top of the basal bulb, the Bourgoin’s organ can be observed ( Figure 3A ), surrounded by three conspicuous blunt-tipped peg-like styloconic sensilla, dome-like processes ( Figure 3C ), and cuticular microdigitations ( Figure 3D ). The distal part of the basal bulb gives rise to a long, thread-like arista and ends with a sharp apex ( Figure 2A ).

### Types and distribution of the antennal and maxillary sensilla

SEM images show eight major types of sense organs on the antennae and maxillae: trichoid sensilla, plate organs, campaniform sensilla, coeloconic sensilla, styloconic sensilla, Bourgoin’s organ, button-like sensilla, and Evans’ organ. Details of each sensillum or organ are described below.

### Trichoid sensilla (Tr).

**Figure 4.**
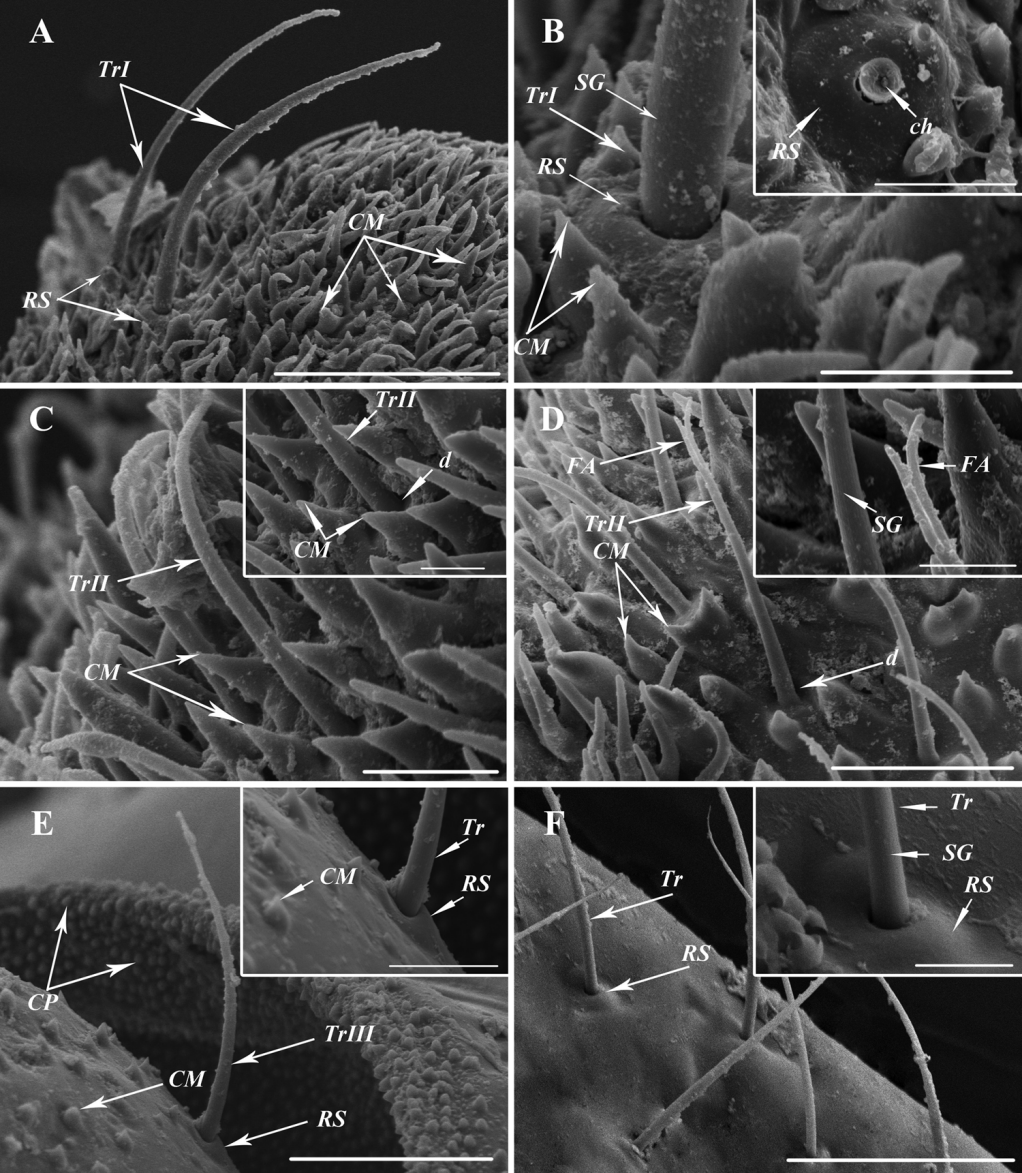
SEM images of trichoid sensilla of *Cixiopsis punctatus.* A, B: Trichoid sensilla subtype I (TrI) on the antennal pedicel. C, D: Ttrichoid sensilla subtype II (TrII) on the antennal pedicel. E: Trichoid sensilla subtype III (TrIII) on the antennal scape. F: Trichoid sensilla (Tr) on the maxilla. ch, channel; CM, cuticular microtubercles; CP, cone-shaped processes; d, depression; FA, forked apex; R.S, raised socket; SG, straight grooves. [(A) scale bar 50 **|x** m, (B) scale bar 10|xm, 10 **|x** m in box, (C) scale bar 10 **|x** m, 5 **|x** m in box, (D) scale bar 20 **|x** m, 5 **|x** m in box, (E) scale bar 30 **|x** m, 10 **|x** m in box, (F) scale bar 30 **|x** m, 5 **|x** m in box]. High quality figures are available online.

Tr are common on the antennal and the maxillary surfaces of insects. The Tr on antennae can be divided into three types, referred to here as TrI, TrII, and TrIII. TrI ( Figure 2A , B) are bristle-like and scattered on the surface of the antennal pedicel. They are 46-96 µm long, 3.0-3.2 µm in basal diameter, with blunt tip and straight longitudinal grooves on the surfaces ( Figure 4A , B). Each TrI is found inserted into an evident raised socket and protrude 30-45° from the antenna ( Figure 4A , B). TrII ( Figure 4C , D) of 28-37 µm length are usually limited on the antennal pedicel and about 2.4 µm in basal diameter. They are blunt-tipped and curved towards the antennal shaft ( Figure 4C ) and this type is occasionally observed with forked apex ( Figure 4D ). Each of them have straight longitudinal patterns and are inserted into a depression, which is about 5.2 µm in diameter ( Figure 4C , D). TrIII (Figures 2A, 4E) are distributed on the base of the antennal scape (21-49 µm long, 2.5 µm in basal diameter). This type, which might be the Böhm bristle, morphologically resembles TrI, but is smaller in size.

In addition, Tr are widely distributed on the maxillae (48-74 µm long, 1.9 µm in basal diameter, Figures 2D, 4F). Similar to TrI, they insert into raised sockets and have straight longitudinal grooves on their surfaces ( Figure 4F ).

### Plate organs (PO).

Setalike PO were founded in *C. punctatus* of both sexes (Figures 2B, C, 3B, E). They are scattered on the antennal pedicel (Figures 2B, 2C, 3B). Each PO consists of approximately 10 setalike projections encircled by rings of cuticular denticles, which are shorter than those projections or as long as them ( Figure 3E ). These setalike projections are solid inside ( Figure 3F ), curved concentrically, and tapering from flat bases into blunt tips ( Figure 3E , F). The cuticular microtubercles (CM) are randomly distributed around, not part of the PO, and not circularly arranged ( Figure 3E ).

### Campaniform sensilla (Ca).

Ca are very few in number, usually located on the antennal and the maxillary surfaces, which are ubiquitous in insects. Based on the position, Ca can be distinguished as CaI, CaII, CaIII. A single CaI ( Figure 2C ) (8.1-10.0 µm in diameter) is presented on the apical surface of the antennal pedicel. CaI is a dome-shaped structure located in a cavity, which is surrounded by thick walls and cone-shaped projections ( Figure 2C ). Only one of the CaII ( Figure 2B ) (17-19 µm in diameter) is founded on the antennal scape. Different from the CaI, CaII rise up from the surface and are surrounded by cuticular microtubercles ( Figure 2B ). A CaIII ( Figure 2D ) (15-17 µm in diameter), similar to CaII, occurs on the maxilla, with 106 µm away from the base of antennal scape.

### Bourgoin’s organ (BO).

On the top of bulb base of the antennal flagellum, there is an evident BO with an elliptical aperture (about 8 µm in long shaft, about 4 µm in short shaft) ( Figure 3A , B, C). The aperture is surrounded by denticlelike walls with 5-11 µm of height ( Figure 3C ). Next to the denticlelike walls, three blunt-tipped, peg-like styloconic sensilla (St, 1µm in diameter, 2-3 µm in height) of triangular arrangement were identified ( Figure 3C , D). Additionally, two dome-like processes are separately distributed near the denticlelike walls ( Figure 3C ), and some cuticular spines are located on BO’s surface ( Figure 3D ). Coeloconic sensilla (Co) are usually shielded by BO, and one of them can be seen directly here ( Figure 3C ).

### Evans’ organ (EO).

A single EO (29–34 µm in diameter) is detected at each geno-maxillar sulcus, with a distance of roughly 170 µm from the base of the antennal scape ( Figure 2D ). Each EO is formed by a deep cavity, which is surrounded by cone-shaped projections and has four petal-like infoldings inside ( Figure 2E ).

### Button-like sensilla (Bl).

Six Bl (2–9 µm in diameter) are discovered on the maxilla near the antennal scape ( Figure 2F ). This type of sensory equipments is composed of a deep cavity with a raised, irregular plate in the center, looking like a button ( Figure 2F ).

### Gross morphology and sensilla of the labium

**Figure 5.**
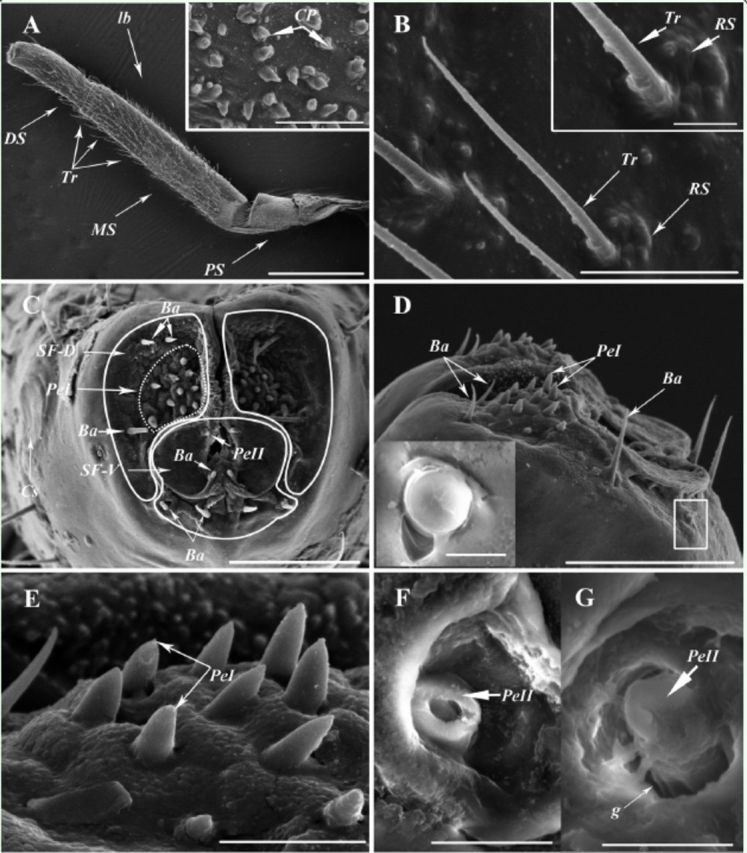
SEM images of the labium of *Cixiopsis punctatus.* A: General view of the labium (lb) with trichoid sensilla (Tr), showing the distal segment (DS), middle segment (MS), proximal segment (PS) and cone-shaped processes (CP) on the proximal segment. B: Trichoid sensilla (Tr) on the distal segment. C: Labial tip, showing the sensory fields with the basiconic sensilla (Ba), peg sensilla (PeI, PeII), and coin-shaped sensilla (Cs). D: The top of the distal segment, showing the basiconic sensilla (Ba) and peg sensilla (PeI). E: Enlarged view of the hollow peg sensilla (PeI). F and G: Enlarged view of the PeII located between the dorsal sensory field (SF-D) and the opening of the maxillae and mandibles, showing the hollow inside and grooves (g) on the surfaces. RS, raised socket; SF-V ventral sensory field. [(A) scale bar 500 **|x** m, 20 **|x** m in box, (B) scale bar 20 **|x** m, 5 **|x** m in box, (C) scale bar 50 **|x** m, (D) scale bar 50 **|x** m, 2 **|x** m in box, (E) scale bar 10 **|x** m, (F) scale bar 5 **|x** m, (G) scale bar 4 |xm]. High quality figures are available online.

Every *C. punctatus* has a three-segmented labium, which is highly adapted to piercing and sucking, including a shortest proximal segment, the longest middle segment, and the shorter distal segment ( Figure 5A ). The outer mandibular and the inner maxillary mouthparts form the stylet bundle, which lies within a groove in the labium. Dorsal sensory field concave is more extended and reaches laterally to the mandibular and maxillary stylets. Four types of sensilla are observed on the labium: trichoid sensilla (Tr), peg sensilla (Pe), basiconic sensilla (Ba), and coin-shaped sensilla (Cs). In addition, cone-shaped processes (CP, 1.8–5.5 µm in height, 1.5–4.0 µm in basal diameter) are widely distributed on the surface of the proximal segment ( Figure 5A ). Above the ventral sensory field (SF-V), there is a pair of basiconic sensilla (PeII, 2.2–3.0 µm in diameter) placed slightly between the dorsal sensory field (SF-D) and the maxillary and mandibulary stylets ( Figure 5C , F, G).

### Tritroid sensilla (Tr).

Tr can be found on the middle and the distal segment ( Figure 5A , B). On the back and lateral surface of the middle segment, Tr (66.1–94.6 µm in length, 2.8–4.3 µm in basal diameter) are inserted into evident sockets, bending towards the labial apex. On the ventral surface of the middle segment, two rows of Tr (about 29.0 µm in length) are located on both side of the labial groove, inserting into shallow pits. The Tr (46.1–116.9 in length, 1.9–2.7 µm in basal diameter) scattered on the distal segment curve towards the distal part, insert into unobvious sockets, and have straight longitudinal grooves on the surface ( Figure 5B ).

### Peg sensilla (Pe).

Two types of Pe are identified on the labial apex, being regarded as PeI and PeII. Each dorsal sensory field on each lateral lobe possesses a terminal field of 10 PeI, varying in length from 2.1–6.2 µm with a basal diameter of 1.8–2.9 µm ( Figure 5C , D, E). They are inserted in depression on wrinkled surface of labial tip, with smooth surface and sharp apex and hollow inside ( Figure 5C , D, E). Two PeII (3.2–4.3 µm in length, 1.8– 2.6 µm in basal diameter) located between the SF-D and the opening of the maxillae and mandibles ( Figure 5C , F, G).

### Basiconic sensilla (Ba).

Five pairs of Ba (10.3–23.3 µm in length, 1.4–2.2 µm in basal diameter) are located in apex of labium, of which three pairs are located in the SF-D and two pairs situated below the SF-V ( Figure 5C , D).

### Coin-shaped sensilla (Cs).

Two Cs are identified on the lateral surfaces of the distal segment, with one at each side. Each of them is located with 116.9–124.6 µm away from the labial apex, and its diameter is measured as 18.4–22.2 µm ( Figure 5C ).

## Discussion

The antennal morphology of *C. punctatus* is similar to that in other fulgoromorphan species. Trichoid sensilla III (TrIII) on the antennal scape ( Figure 4E ) are similar to Böhm bristles, which are present in analogous locations in various insects ( Heran 1959 ; Markl 1962 ; Schneider 1964 ) and might function as mechanoreceptors or proprioceptors ( Pringle 1938 ; Thurm 1962 ; Schneider 1964 ; Sane et al. 2007 ).

Bourgoin's organ (BO) seems to be present in all fulgoromorphan families, including an aperture surrounded by a ridge on the top, coeloconic sensilla (Co) inside, and sometimes styloconic sensilla (St) (in Cixiidae, Achilixiidae) beside the ridge ( Bourgoin 1985 ; Cobben 1988 ; Shih and Yang 1996 ; Liang 2001 ; Liang and Fletcher 2002 ; Romani et al. 2009 ). The ridge has been revealed as three different types: single ring, petal-like wall *(Kallitaxila granulata, Zema gressitti* in Tropiduchidae), fringed or digitate wall (Achilixiidae, Derbidae, Meenoplidae, Kinnaridae, Tettigometridae, Ricaniidae, part of Cixiidae and Flatidae) ( Bourgoin 1985 ; Shih and Yang 1996 ; Liang 2001 ; Romani et al. 2009 ; Wang et al. 2012 , 2013). In *C. punctatus,* the ridge is single ring type with several denticles on it, and a Co can be identified easily through the aperture ( Figure 3C ). The morphology of St ( Figure 3C , D) in *C. punctatus* is consistent with those in previous records and is the first found in Tropiduchidae.

Campaniform sensilla (Ca) are present in various places, usually near the segmental joints on insects, such as halteres, palps, legs, bases of wings, and eyes ( Schneider 1964 ; Bromley et al. 1980 ), and have been reported in Fulgoroidea as well ( Schneider 1964 ; Bourgoin 1985 ; Romani et al. 2009 ; Bartlett and Hamilton 2011 ). However, only a few cases of campaniform sensilla II (CaII) are found on the antennal scape ( Bartlett and Hamilton 2011 ), and campaniform sensilla III (CaIII) on the maxillae have rarely been discovered, except for in *Kallitaxila granulata* and *Z. gressitti* ( Wang et al. 2012 , 2013). In addition, comparing with *K. granulata* and *Z. gressitti,* with three campaniform sensilla III (CaIII) ( Wang et al. 2012 , 2013), *C. punctatus* only have one campaniform sensilla III (CaIII) on each side of maxillae ( Figure 2D ).

Plate organs (PO) in Fulgoromorpha, with structural variations, are divided into five main morphological types, two types of which have been reported in Tropiduchidae: the setalike projected and the folded flattened plate (often clover leaf-like) ( Bourgoin and Deiss 1994 ; Wang et al. 2012 , 2013). The PO of *C. punctatus* are in setalike projected form ( Figure 3E , F), similar to some tropichuchid species, e.g. *Kusuma* sp. ( Marshall and Lewis 1971 ), *Trypetimorpha japonica* ( Huang and Bourgoin 1993 ), *Teramnon stenopteryx* (Hamilton 2011), and *Z. gressitti* ( Wang et al. 2013 ). However, they are different from those with a folded flattened form in *Ossoides line-atus* and *K. granulata* in Tropiduchidae, *Microflata stictica* in Flatoidae, and *Lophops carinatus* in Lophopidae ( Marshall and Lewis 1971 ; Bourgoin and Deiss 1994 ; Stroihski et al. 2011; Wang et al. 2012).

Evans’ organ (EO) were first reported as ‘a finger-like lobe contained in a pit’ in Auchenorrhyncha by Evans (1973) , and are considered to be important in understanding the origin of the head capsule in Hemiptera ( Evans 1973 ; Bourgoin 1986 ). The position of EO on the maxillary plates varies according to the taxa: dorsally or ventrally to the maxillary sulcus when present, very posteriorly on the gena, or very anteriorly under the antennal socket ( Bourgoin 1986 ). Additionally, EO was named as ‘subantennal plaque sensillum’ on the subantennal process in *Borysthenes maculata* and *Euryphlepsia papuaensis* of Cixiidae ( Liang 2005a ). In *C. punctatus,* its position resembles that observed in most fulgoromorpha taxa, such as *Z. gressitti* of Tropiduchidae, at the basal marge of the gena ( Figure 2D ). EO is apparently absent in Sternorrhyncha and Heteroptera, whereas it is present in Coleorrhyncha as a placoid-like sensillum ( Bourgoin 1986 ).

Peg sensilla (Pe) on labial tip in *C. punctatus* are quite common on the labium in other Fulgoromorpha, e.g., *Nilaparvata lugens* (Delphacidae) ( Foster et al. 1983 : Figure 1b ) and *Andes marmorata* (Cixiidae) ( Liang 2005b : Figure 2A ), and they vary in number. Brożek and Bourgoin (2012) regarded peg sensilla I (PeI) as uniporous peg sensilla (PGSU1/2) and multiporous peg sensilla (PGSM), and basiconic sensilla (Ba) as long sensilla basiconica (BSN1). However, to accurately define the uniporous peg sensilla (PGSU1/2) and multiporous peg sensilla (PGSM) in *C. punctatus,* further study on the inner structure of Peg sensilla I (PeI) is still necessary. What make this species special are the distinct structures and new morphological characteristics. Peg sensilla II (PeII) were discovered in the tropiduchid labium. The location of PeII ( Figure 5C , F, G) is similar to the region of oval plate sensillum, multiporous (OPSM), described in *Nogodina reticulata* (Nogodinidae), and a similar position of the long sensilla basiconica (BSN1) is also found in Lophopidae ( Brożek and Bourgoin 2012 ). The BSN1 in Lophopidae is supposed to represent a specialized pattern (lophopid pattern) that has probably evolved from the issid one ( Brożek and Bourgoin 2012 ). The distribution patterns of the sensilla on the labial tip therefore may contribute to the clarification of evolutionary relationship within Fulgoromorpha.

We found coin-shaped sensilla (Cs) in *C. punctatus* on the distal labial segment, similar to *Z. gressitti, K. granulata,* and *Lavora ricanoides* of Tropiduchidae (Rong-rong Wang, unpublished data). Cs correspond to special sensory organs known as subapical sensory organs ( Backus 1985 ) and latero-subapical labial sensilla ( Liang 2005b ), and have been reported in other planthoppers taxa in multifarious forms: peg-like in *Borysthenes maculata* and *Andes marmorata* of Cixiidae ( Liang 2005b ), multilobed in *Nilaparvata lugens* and other Delphacidae ( Foster et al. 1983 : Figure 1a ; Sōgawa 1981 ). Moreover, Brożek and Bourgoin (2012) named several different types of these analogous sensilla.

## Abbreviations

Ba: basiconic sensilla
Bl: button-like sensilla
BO: Bourgoins organ
BSN1: sensillum basiconicum, nonporous, long
Ca: campaniform sensilla
CM: uticular microtubercle
Co: coeloconic sensillum
CP: cone-shaped process
Cs: coin-shaped sensilla
EO: Evans organ
OPSM: oval plate sensillum, multiporous
Pe: peg sensilla
PGSM: peg sensillum, multiporous
PGSU1: peg sensillum, uniporous, long
PGSU2: peg sensillum, uniporous, short
PO: plate organs
SF-D: dorsal sensory field
SF-V: ventral sensory field
Tr: trichoid sensilla
